# Supplementary material for: Direct selection of functional fluorescent-protein antibody fusions by yeast display
Source: PLoS One. 2023 Feb 24;18(2):e0280930. doi: 10.1371/journal.pone.0280930 (PMC9956592; doi:10.1371/journal.pone.0280930)
Supplement: S1 Fig — The chromatograms obtained for the four scTGPs are shown in comparison to the molecular weight marker. (DOCX) [file pone.0280930.s001.docx]

**Supporting Information**

**S1 Figure: Chromatograms.**

The chromatograms obtained for the four scTGPs are shown in comparison to the molecular weight marker.
