# Supplementary material for: Direct selection of functional fluorescent-protein antibody fusions by yeast display
Source: PLoS One. 2023 Feb 24;18(2):e0280930. doi: 10.1371/journal.pone.0280930 (PMC9956592; doi:10.1371/journal.pone.0280930)
Supplement: S1 Table — (DOCX) [file pone.0280930.s002.docx]

**S1Table: DNA and amino acid sequences of the clones expressed and purified as scFvs and scTGP.**

| **clone** | **scFv_DNA** | **scTGP_DNA** | **scFv_AA** | **scTGP_AA** |
| --- | --- | --- | --- | --- |
| CDK2A-A8 | GAAATTGTATTGACGCTGACTCAGCCACCCTCGGTGTCAGTGGCCCCAGGACAGACGGCCAGGATTACCTGTGGTGGAAACAACATTGGAAGTAAAAGTGTGCACTGGTACCAGCAGAAGCCAGGCCAGGCCCCTGTGCTGGTCGTCTATGATGATAGCGACCGGCCCTCAGGGATCCCTGAGCGATTCTCTGGCTCCAACTCTGGGAACACGGCCACCCTGACCATCAGCAGGGTCGAAGCCGGGGATGAGGCCGACTATTACTGTCAGGTGTGGGATAGTAGTAGTGATCCTTATGTCTTCGGAACTGGGACCAAGCTCACCGTCCTATCTtccggagggtcgaccataacttcgtataatgtatactatacgaagttatcctcgagcggtaccACCCAGGTCCAGCTTGTGCAGTCTGGAGCAGAGGTGAAAAAGCCGGGGGAGTCTCTGAAGATCTCCTGTAAGGGTTCTGGATACAGCTTTACCAGCTACTGGATCGGCTGGGTGCGCCAGATGCCCGGGAAAGGCCTGGAGTGGATGGGGATCATTTATCCTGGTGACTCTGATACCAGGTACAGCCCGTCCTTCCAAGGCCAGGTCACCATCTCAGCCGACAAGTCCAGCAGCACCGTCTACCTGCAGTGGAGCAGCCTGACGGCCTCGGACACCGCCATGTATTACTGTGCGAGCCAGGGCTTTCAGGGAGATGCTTTTGATATCTGGGGCCAAGGGACAATGGTACCGTCTCTTCA | GAAATTGTATTGACGCTGACTCAGCCACCCTCGGTGTCAGTGGCCCCAGGACAGACGGCCAGGATTACCTGTGGTGGAAACAACATTGGAAGTAAAAGTGTGCACTGGTACCAGCAGAAGCCAGGCCAGGCCCCTGTGCTGGTCGTCTATGATGATAGCGACCGGCCCTCAGGGATCCCTGAGCGATTCTCTGGCTCCAACTCTGGGAACACGGCCACCCTGACCATCAGCAGGGTCGAAGCCGGGGATGAGGCCGACTATTACTGTCAGGTGTGGGATAGTAGTAGTGATCCTTATGTCTTCGGAACTGGGACCAAGCTCACCGTCCTATCTTCTGGAGGGTCGACCATAACTTCGGTAATTAAACCGGAAATGAAAATTAAATTGCGTATGGAAGGTGCCGTTAACGGCCATAAATTTGTAATTGAAGGAGAAGGAATAGGCAAACCATACGAAGGAACCCAGACCCTGGATTTAACCGTAGAAGAAGGCGCACCTCTCCCTTTCTCGTACGACATCCTCACCCCAGCCTTCCAATACGGCAATCGCGCTTTCACCAAATACCCAGAAGATATTCCAGACTATTTTAAACAAGCATTCCCCGAAGGCTATTCTTGGGAACGCTCTATGACCTATGAAGATCAAGGAATTTGTATCGCTACTTCCGACATTACTATGGAAGGAGACTGTTTTTTTTATGAAATTCGCTTTGATGGAACTAACTTCCCCCCGAACGGCCCTGTAATGCAAAAGAAGACCTTAAAATGGGAACCTAGCACCGAAAAAATGTATGTAGAAGACGGAGTTCTTAAGGGTGACGTAGAAATGGCACTTCTGCTCGAAGGAGGTGGACACTACCGCTGCGATTTTAAAACCACTTATAAAGCCAAAAAAGATGTTCGTCTTCCAGATGCACACGAGGTGGACCACCGCATTGAAATCCTGAGCCACGATAAAGATTATAATAAAGTTAGACTCTATGAACACGCCGAAGCCCGCTATTCTGGCGGAGGCAGCGGATCCTCAAGCGGTACCCAGGTCCAGCTTGTGCAGTCTGGAGCAGAGGTGAAAAAGCCGGGGGAGTCTCTGAAGATCTCCTGTAAGGGTTCTGGATACAGCTTTACCAGCTACTGGATCGGCTGGGTGCGCCAGATGCCCGGGAAAGGCCTGGAGTGGATGGGGATCATTTATCCTGGTGACTCTGATACCAGGTACAGCCCGTCCTTCCAAGGCCAGGTCACCATCTCAGCCGACAAGTCCAGCAGCACCGTCTACCTGCAGTGGAGCAGCCTGACGGCCTCGGACACCGCCATGTATTACTGTGCGAGCCAGGGCTTTCAGGGAGATGCTTTTGATATCTGGGGCCAAGGGACAATGGTCACCGTCTCTTCA | EIVLTLTQPPSVSVAPGQTARITCGGNNIGSKSVHWYQQKPGQAPVLVVYDDSDRPSGIPERFSGSNSGNTATLTISRVEAGDEADYYCQVWDSSSDPYVFGTGTKLTVLSSGGSTITSYNVYYTKLSSSGTTQVQLVQSGAEVKKPGESLKISCKGSGYSFTSYWIGWVRQMPGKGLEWMGIIYPGDSDTRYSPSFQGQVTISADKSSSTVYLQWSSLTASDTAMYYCASQGFQGDAFDIWGQGTMVTVSS | EIVLTLTQPPSVSVAPGQTARITCGGNNIGSKSVHWYQQKPGQAPVLVVYDDSDRPSGIPERFSGSNSGNTATLTISRVEAGDEADYYCQVWDSSSDPYVFGTGTKLTVLSSGGSTITSVIKPEMKIKLRMEGAVNGHKFVIEGEGIGKPYEGTQTLDLTVEEGAPLPFSYDILTPAFQYGNRAFTKYPEDIPDYFKQAFPEGYSWERSMTYEDQGICIATSDITMEGDCFFYEIRFDGTNFPPNGPVMQKKTLKWEPSTEKMYVEDGVLKGDVEMALLLEGGGHYRCDFKTTYKAKKDVRLPDAHEVDHRIEILSHDKDYNKVRLYEHAEARYSGGGSGSSSGTQVQLVQSGAEVKKPGESLKISCKGSGYSFTSYWIGWVRQMPGKGLEWMGIIYPGDSDTRYSPSFQGQVTISADKSSSTVYLQWSSLTASDTAMYYCASQGFQGDAFDIWGQGTMVTVSS |
| CDK2A-F2 | GAAATTGTATTGACGCAGTCTCCATCCTCCCTGTCTGCATCTGTAGGAGACAGAGTCACCATCACTTGCCAGGCGAGTCAGGACATTGACACCTATTTAAATTGGTATCAGCAGAAACCAGGGAAAGCCCCTAAACTCCTGATCTACGATGCATCTAATTTGGAGACAGGGGTCCCATCAAGGTTCAATGGAGGCGGATCTGGGACAGATTTTAGTTTGACCATCAGTAGCCTGCAGCCTGAAGATATTGCAACATATTACTGTCAACAATATGACAGTCTCCCGATCACCTTCGGCCAAGGGACACGACTGGAGATTAAAtccggagggtcgaccataacttcgtataatgtatactatacgaagttatcctcgagcggtaccACCCAGGTCCAGCTGGTACAGTCTGGGGCTGAGGTGAAGAAGCCTGGGGCCTCAGTGAAGGTCTCCTGCAAGGCTTCTGGATACACCTTCACCGGCTACTATATGCACTGGGTGCGACAGGCCCCTGGACAAGGGCTTGAGTGGATGGGAATAATCAACCCTAGTGGTGGTAGCACAAGCTACGCACAGAAGTTCCAGGGCAGAGTCACCATGACCAGGGACACGTCCACGAGCACAGTCTACATGGAGCTGAGCAGCCTGAGATCTGAGGACACGGCCGTGTATTACTGTGCTAGAGGAACTGAGGGCTGGTTCGACCCCTGGGGCCAGGGAACCCTGGTCACCGTCTCCTCA | GAAATTGTATTGACGCAGTCTCCATCCTCCCTGTCTGCATCTGTAGGAGACAGAGTCACCATCACTTGCCAGGCGAGTCAGGACATTGACACCTATTTAAATTGGTATCAGCAGAAACCAGGGAAAGCCCCTAAACTCCTGATCTACGATGCATCTAATTTGGAGACAGGGGTCCCATCAAGGTTCAATGGAGGCGGATCTGGGACAGATTTTAGTTTGACCATCAGTAGCCTGCAGCCTGAAGATATTGCAACATATTACTGTCAACAATATGACAGTCTCCCGATCACCTTCGGCCAAGGGACACGACTGGAGATTAAATCTGGAGGGTCGACCATAACTTCGGTAATTAAACCGGAAATGAAAATTAAATTGCGTATGGAAGGTGCCGTTAACGGCCATAAATTTGTAATTGAAGGAGAAGGAATAGGCAAACCATACGAAGGAACCCAGACCCTGGATTTAACCGTAGAAGAAGGCGCACCTCTCCCTTTCTCGTACGACATCCTCACCCCAGCCTTCCAATACGGCAATCGCGCTTTCACCAAATACCCAGAAGATATTCCAGACTATTTTAAACAAGCATTCCCCGAAGGCTATTCTTGGGAACGCTCTATGACCTATGAAGATCAAGGAATTTGTATCGCTACTTCCGACATTACTATGGAAGGAGACTGTTTTTTTTATGAAATTCGCTTTGATGGAACTAACTTCCCCCCGAACGGCCCTGTAATGCAAAAGAAGACCTTAAAATGGGAACCTAGCACCGAAAAAATGTATGTAGAAGACGGAGTTCTTAAGGGTGACGTAGAAATGGCACTTCTGCTCGAAGGAGGTGGACACTACCGCTGCGATTTTAAAACCACTTATAAAGCCAAAAAAGATGTTCGTCTTCCAGATGCACACGAGGTGGACCACCGCATTGAAATCCTGAGCCACGATAAAGATTATAATAAAGTTAGACTCTATGAACACGCCGAAGCCCGCTATTCTGGCGGAGGCAGCGGATCCTCAAGCGGTACCCAGGTCCAGCTGGTACAGTCTGGGGCTGAGGTGAAGAAGCCTGGGGCCTCAGTGAAGGTCTCCTGCAAGGCTTCTGGATACACCTTCACCGGCTACTATATGCACTGGGTGCGACAGGCCCCTGGACAAGGGCTTGAGTGGATGGGAATAATCAACCCTAGTGGTGGTAGCACAAGCTACGCACAGAAGTTCCAGGGCAGAGTCACCATGACCAGGGACACGTCCACGAGCACAGTCTACATGGAGCTGAGCAGCCTGAGATCTGAGGACACGGCCGTGTATTACTGTGCTAGAGGAACTGAGGGCTGGTTCGACCCCTGGGGCCAGGGAACCCTGGTCACCGTCTCCTCA | EIVLTQSPSSLSASVGDRVTITCQASQDIDTYLNWYQQKPGKAPKLLIYDASNLETGVPSRFNGGGSGTDFSLTISSLQPEDIATYYCQQYDSLPITFGQGTRLEIKSGGSTITSYNVYYTKLSSSGTTQVQLVQSGAEVKKPGASVKVSCKASGYTFTGYYMHWVRQAPGQGLEWMGIINPSGGSTSYAQKFQGRVTMTRDTSTSTVYMELSSLRSEDTAVYYCARGTEGWFDPWGQGTLVTVSS | EIVLTQSPSSLSASVGDRVTITCQASQDIDTYLNWYQQKPGKAPKLLIYDASNLETGVPSRFNGGGSGTDFSLTISSLQPEDIATYYCQQYDSLPITFGQGTRLEIKSGGSTITSVIKPEMKIKLRMEGAVNGHKFVIEGEGIGKPYEGTQTLDLTVEEGAPLPFSYDILTPAFQYGNRAFTKYPEDIPDYFKQAFPEGYSWERSMTYEDQGICIATSDITMEGDCFFYEIRFDGTNFPPNGPVMQKKTLKWEPSTEKMYVEDGVLKGDVEMALLLEGGGHYRCDFKTTYKAKKDVRLPDAHEVDHRIEILSHDKDYNKVRLYEHAEARYSGGGSGSSSGTQVQLVQSGAEVKKPGASVKVSCKASGYTFTGYYMHWVRQAPGQGLEWMGIINPSGGSTSYAQKFQGRVTMTRDTSTSTVYMELSSLRSEDTAVYYCARGTEGWFDPWGQGTLVTVSS |
| USP11-B6 | GAAATTGTGATGACCCAGACTCCAGGCACCCTGTGCTTGTCTCCAGGGGAAAGAGCCACCCTCTCCTGCAGGGCCAGTCAGAGTGTTAGCAACAACAACTTAGCCTGGTACCAGCAGAAACCTGGCCAGGCTCCCAGGCTCCTCATCTTTGGTGCATCCAACAGGGCCACTGGCATCCCAGACAGGTTTAGTGGCAGTGGGTCTGGGACAGACTTCACTCTCACTATCAGCAGCCTCCAGTCTGAAGATTTTGCAGTTTATTACTGTCAGCAGTATGATAGTTGGCCCCTTACTTTCGGCGGGGGGACCAAGCTGGAGATCAAAtccggagggtcgaccataacttcgtataatgtatactatacgaagttatcctcgagcggtaccACCCAGGTCCAGCTGGTGCAGTCTGGGGCTGAGGTGAAGAAGCCTGGGTCCTCGGTGAAGGTCTCCTGCAAGGCTTCTGGAGGCACCTTCAGCAGCTATGCTATCAGCTGGGTGCGACAGGCCCCTGGACAAGGGCTTGAGTGGATGGGAGGGATCATCCCTATCTTTGGTACAGCAAACTACGCACAGAAGTTCCTGGGCAGAGTCACGATTACCGCGGACGAATCCACGAGCACAGCCTACATGGAGCTGAGCAGCCTGAGATCTGAGGACACGGCCGTGTATTACTGTGCGAGAGAAGGGGGTATAGGGTTGTCCGGCTGGCTCGACCCCTGGGGCCAGGGAACCCTGGTCACTGTCTCCTCA | GAAATTGTGATGACCCAGACTCCAGGCACCCTGTGCTTGTCTCCAGGGGAAAGAGCCACCCTCTCCTGCAGGGCCAGTCAGAGTGTTAGCAACAACAACTTAGCCTGGTACCAGCAGAAACCTGGCCAGGCTCCCAGGCTCCTCATCTTTGGTGCATCCAACAGGGCCACTGGCATCCCAGACAGGTTTAGTGGCAGTGGGTCTGGGACAGACTTCACTCTCACTATCAGCAGCCTCCAGTCTGAAGATTTTGCAGTTTATTACTGTCAGCAGTATGATAGTTGGCCCCTTACTTTCGGCGGGGGGACCAAGCTGGAGATCAAAGACTATTACTGTCAGGTGTGGGATAGTAGTAGTGATCCTTATGTCTTCGGAACTGGGACCAAGCTCACCGTCCTATCTTCTGGAGGGTCGACCATAACTTCGGTAATTAAACCGGAAATGAAAATTAAATTGCGTATGGAAGGTGCCGTTAACGGCCATAAATTTGTAATTGAAGGAGAAGGAATAGGCAAACCATACGAAGGAACCCAGACCCTGGATTTAACCGTAGAAGAAGGCGCACCTCTCCCTTTCTCGTACGACATCCTCACCCCAGCCTTCCAATACGGCAATCGCGCTTTCACCAAATACCCAGAAGATATTCCAGACTATTTTAAACAAGCATTCCCCGAAGGCTATTCTTGGGAACGCTCTATGACCTATGAAGATCAAGGAATTTGTATCGCTACTTCCGACATTACTATGGAAGGAGACTGTTTTTTTTATGAAATTCGCTTTGATGGAACTAACTTCCCCCCGAACGGCCCTGTAATGCAAAAGAAGACCTTAAAATGGGAACCTAGCACCGAAAAAATGTATGTAGAAGACGGAGTTCTTAAGGGTGACGTAGAAATGGCACTTCTGCTCGAAGGAGGTGGACACTACCGCTGCGATTTTAAAACCACTTATAAAGCCAAAAAAGATGTTCGTCTTCCAGATGCACACGAGGTGGACCACCGCATTGAAATCCTGAGCCACGATAAAGATTATAATAAAGTTAGACTCTATGAACACGCCGAAGCCCGCTATTCTGGCGGAGGCAGCGGATCCTCAAGCGGTACCCAGGTCCAGCTGGTGCAGTCTGGGGCTGAGGTGAAGAAGCCTGGGTCCTCGGTGAAGGTCTCCTGCAAGGCTTCTGGAGGCACCTTCAGCAGCTATGCTATCAGCTGGGTGCGACAGGCCCCTGGACAAGGGCTTGAGTGGATGGGAGGGATCATCCCTATCTTTGGTACAGCAAACTACGCACAGAAGTTCCTGGGCAGAGTCACGATTACCGCGGACGAATCCACGAGCACAGCCTACATGGAGCTGAGCAGCCTGAGATCTGAGGACACGGCCGTGTATTACTGTGCGAGAGAAGGGGGTATAGGGTTGTCCGGCTGGCTCGACCCCTGGGGCCAGGGAACCCTGGTCACTGTCTCCTCA | EIVMTQTPGTLCLSPGERATLSCRASQSVSNNNLAWYQQKPGQAPRLLIFGASNRATGIPDRFSGSGSGTDFTLTISSLQSEDFAVYYCQQYDSWPLTFGGGTKLEIKSGGSTITSYNVYYTKLSSSGTTQVQLVQSGAEVKKPGSSVKVSCKASGGTFSSYAISWVRQAPGQGLEWMGGIIPIFGTANYAQKFLGRVTITADESTSTAYMELSSLRSEDTAVYYCAREGGIGLSGWLDPWGQGTLVTVSS | EIVMTQTPGTLCLSPGERATLSCRASQSVSNNNLAWYQQKPGQAPRLLIFGASNRATGIPDRFSGSGSGTDFTLTISSLQSEDFAVYYCQQYDSWPLTFGGGTKLEIKDYYCQVWDSSSDPYVFGTGTKLTVLSSGGSTITSVIKPEMKIKLRMEGAVNGHKFVIEGEGIGKPYEGTQTLDLTVEEGAPLPFSYDILTPAFQYGNRAFTKYPEDIPDYFKQAFPEGYSWERSMTYEDQGICIATSDITMEGDCFFYEIRFDGTNFPPNGPVMQKKTLKWEPSTEKMYVEDGVLKGDVEMALLLEGGGHYRCDFKTTYKAKKDVRLPDAHEVDHRIEILSHDKDYNKVRLYEHAEARYSGGGSGSSSGTQVQLVQSGAEVKKPGSSVKVSCKASGGTFSSYAISWVRQAPGQGLEWMGGIIPIFGTANYAQKFLGRVTITADESTSTAYMELSSLRSEDTAVYYCAREGGIGLSGWLDPWGQGTLVTVSS |
| USP11-C3 | GAAATTGTGTTGACACAGTCTCCAGGCACCCTGTCTTTGTCTCCAGGGGAGAGAGCTACCCTCTTTTGCAGGGCCAGTCAGAGTGTTAGCAGCAACTTAGCCTGGTACCAGCAAAAACCTGGCCAGGCTCCCAGGCTCCTCATCTATGGTGCATCCACCAGGGCCACTGGTATTCCAGCCAGGTTCAGTGGCAGTGGGTCTGGGACAGAGTTCACTCTCACCATCAGCAGCCTGCAGTCTGAAGATTTTGCAGTTTATTACTGTCAGCAGTATGATGACTGGCCTCTCACTTTCGGCGGAGGGACCAAAGTGGATATCAAAtccggagggtcgaccataacttcgtataatgtatactatacgaagttatcctcgagcggtaccACCCAGGTACAGCTGGTGCAGTCTGGGGCTGAGGTGAAGAAGCCTGGGTCCTCGGTGAAGGTCTCCTGCAAGGCTTCTGGAGGCACCTTCAGCAGCTATGCTATCAGCTGGGTGCGACAGGCCCCTGGACAAGGGCTTGAGTGGATGGGAGGGATCATCCCTATCTTTGGTACAGCAAACTACGCACAGAAGTTCCAGGGCAGAGTCACGATTACCGCGGACGAATCCACGAGCACAGCCTACATGGAGCTGAGCAGCCTGAGATCTGAGGACACGGCCGTGTATTACTGTGCGAGAGAAGGGGGTATAGGGTTGTCCGGCTGGCTCGACCCCTGGGGCCAGGGAACCCTGGTCACCGTCTCCTCA | GAAATTGTGTTGACACAGTCTCCAGGCACCCTGTCTTTGTCTCCAGGGGAGAGAGCTACCCTCTTTTGCAGGGCCAGTCAGAGTGTTAGCAGCAACTTAGCCTGGTACCAGCAAAAACCTGGCCAGGCTCCCAGGCTCCTCATCTATGGTGCATCCACCAGGGCCACTGGTATTCCAGCCAGGTTCAGTGGCAGTGGGTCTGGGACAGAGTTCACTCTCACCATCAGCAGCCTGCAGTCTGAAGATTTTGCAGTTTATTACTGTCAGCAGTATGATGACTGGCCTCTCACTTTCGGCGGAGGGACCAAAGTGGATATCAAAGACTATTACTGTCAGGTGTGGGATAGTAGTAGTGATCCTTATGTCTTCGGAACTGGGACCAAGCTCACCGTCCTATCTTCTGGAGGGTCGACCATAACTTCGGTAATTAAACCGGAAATGAAAATTAAATTGCGTATGGAAGGTGCCGTTAACGGCCATAAATTTGTAATTGAAGGAGAAGGAATAGGCAAACCATACGAAGGAACCCAGACCCTGGATTTAACCGTAGAAGAAGGCGCACCTCTCCCTTTCTCGTACGACATCCTCACCCCAGCCTTCCAATACGGCAATCGCGCTTTCACCAAATACCCAGAAGATATTCCAGACTATTTTAAACAAGCATTCCCCGAAGGCTATTCTTGGGAACGCTCTATGACCTATGAAGATCAAGGAATTTGTATCGCTACTTCCGACATTACTATGGAAGGAGACTGTTTTTTTTATGAAATTCGCTTTGATGGAACTAACTTCCCCCCGAACGGCCCTGTAATGCAAAAGAAGACCTTAAAATGGGAACCTAGCACCGAAAAAATGTATGTAGAAGACGGAGTTCTTAAGGGTGACGTAGAAATGGCACTTCTGCTCGAAGGAGGTGGACACTACCGCTGCGATTTTAAAACCACTTATAAAGCCAAAAAAGATGTTCGTCTTCCAGATGCACACGAGGTGGACCACCGCATTGAAATCCTGAGCCACGATAAAGATTATAATAAAGTTAGACTCTATGAACACGCCGAAGCCCGCTATTCTGGCGGAGGCAGCGGATCCTCAAGCGGTACCCAGGTACAGCTGGTGCAGTCTGGGGCTGAGGTGAAGAAGCCTGGGTCCTCGGTGAAGGTCTCCTGCAAGGCTTCTGGAGGCACCTTCAGCAGCTATGCTATCAGCTGGGTGCGACAGGCCCCTGGACAAGGGCTTGAGTGGATGGGAGGGATCATCCCTATCTTTGGTACAGCAAACTACGCACAGAAGTTCCAGGGCAGAGTCACGATTACCGCGGACGAATCCACGAGCACAGCCTACATGGAGCTGAGCAGCCTGAGATCTGAGGACACGGCCGTGTATTACTGTGCGAGAGAAGGGGGTATAGGGTTGTCCGGCTGGCTCGACCCCTGGGGCCAGGGAACCCTGGTCACCGTCTCCTCA | EIVLTQSPGTLSLSPGERATLFCRASQSVSSNLAWYQQKPGQAPRLLIYGASTRATGIPARFSGSGSGTEFTLTISSLQSEDFAVYYCQQYDDWPLTFGGGTKVDIKSGGSTITSYNVYYTKLSSSGTTQVQLVQSGAEVKKPGSSVKVSCKASGGTFSSYAISWVRQAPGQGLEWMGGIIPIFGTANYAQKFQGRVTITADESTSTAYMELSSLRSEDTAVYYCAREGGIGLSGWLDPWGQGTLVTVSS | EIVLTQSPGTLSLSPGERATLFCRASQSVSSNLAWYQQKPGQAPRLLIYGASTRATGIPARFSGSGSGTEFTLTISSLQSEDFAVYYCQQYDDWPLTFGGGTKVDIKDYYCQVWDSSSDPYVFGTGTKLTVLSSGGSTITSVIKPEMKIKLRMEGAVNGHKFVIEGEGIGKPYEGTQTLDLTVEEGAPLPFSYDILTPAFQYGNRAFTKYPEDIPDYFKQAFPEGYSWERSMTYEDQGICIATSDITMEGDCFFYEIRFDGTNFPPNGPVMQKKTLKWEPSTEKMYVEDGVLKGDVEMALLLEGGGHYRCDFKTTYKAKKDVRLPDAHEVDHRIEILSHDKDYNKVRLYEHAEARYSGGGSGSSSGTQVQLVQSGAEVKKPGSSVKVSCKASGGTFSSYAISWVRQAPGQGLEWMGGIIPIFGTANYAQKFQGRVTITADESTSTAYMELSSLRSEDTAVYYCAREGGIGLSGWLDPWGQGTLVTVSS |
